# Efficient reciprocating burrowing with anisotropic origami feet

**DOI:** 10.3389/frobt.2023.1214160

**Published:** 2023-08-02

**Authors:** Sareum Kim, Laura K. Treers, Tae Myung Huh, Hannah S. Stuart

**Affiliations:** ^1^ Embodied Dexterity Group, Department of Mechanical Engineering, University of California Berkeley, Berkeley, CA, United States; ^2^ Department of Electrical and Computer Engineering, University of California Santa Cruz, Santa Cruz, CA, United States

**Keywords:** anisotropy, soft robot, burrowing, granular media, origami

## Abstract

Origami folding is an ancient art which holds promise for creating compliant and adaptable mechanisms, but has yet to be extensively studied for granular environments. At the same time, biological systems exploit anisotropic body forces for locomotion, such as the frictional anisotropy of a snake’s skin. In this work, we explore how foldable origami feet can be used to passively induce anisotropic force response in granular media, through varying their resistive plane. We present a reciprocating burrower which transfers pure symmetric linear motion into directed burrowing motion using a pair of deployable origami feet on either end. We also present an application of the reduced order model granular Resistive Force Theory to inform the design of deformable structures, and compare results with those from experiments and Discrete Element Method simulations. Through a single actuator, and without the use of advanced controllers or sensors, these origami feet enable burrowing locomotion. In this paper, we achieve burrowing translation ratios—net forward motion to overall linear actuation—over 46% by changing foot design without altering overall foot size. Specifically, anisotropic folding foot parameters should be tuned for optimal performance given a linear actuator’s stroke length.

## 1 Introduction

In granular media, locomotion presents unique challenges. Moving through such media involves resistive forces that are an order of magnitude higher than those in other media (Naclerio et al., 2021). Grains can fluidize or jam, making it necessary to adopt appropriate locomotion strategies for efficient and effective advancement. For instance, sandfish can traverse beneath the sand using undulatory motions, which involve dynamic whole-body locomotion (Maladen et al., 2011). Other creatures, such as mole crabs (Dorgan, 2015), use their limbs to dig or swim through the media. Plant roots also demonstrate growing locomotion (Dexter, 1987). These various types of movements have inspired the development of robotic systems, including robotic snakes (Marvi et al., 2014) with sidewinding and flipper-driven (Mazouchova et al., 2013) walking on the surface of the granular media. Underneath the surface of granular media, self-burrowing of a mole crab-inspired robot with legs (Treers et al., 2022), burrowing with underactuated appendages resulting in an asymmetric profile between power and return strokes (Chopra et al., 2023), and growing with a root-like robot (Naclerio et al., 2021) all show successful burrowing. More reviews on bioinspired robotic burrowers can be found in (Wei et al., 2021). Despite each method’s particular advantages, these systems often necessitate complex mechanisms, leading to intricate robot designs. A promising pathway to achieving simpler burrowing robots is to use reciprocating burrowing using a single linear actuator.

Reciprocating burrowers transform oscillatory linear actuation into net directed motion. This reciprocal behavior can be found in natural systems, such as the ovipositors of wood wasps (Vincent and King, 1995) and locusts (Vincent, 1976). By broadening the definition of reciprocating actions, push-and-pull locomotions exhibited by earthworms (Quillin, 1999) or razor clams (Winter et al., 2014) can also be classified as reciprocating burrowers. These reciprocating systems generate net propulsion without net material flow (Purcell, 1977) and offer several advantages (Wei et al., 2021): 1) simple structure and movement, 2) automatic debris discharge, and 3) no need for additional appendage actuators to provide external axial force. Robotic systems employing reciprocating burrowing typically utilize simple motorized or pneumatic linear actuators. However, these systems require another crucial feature: anisotropic force response, or direction-dependent asymmetry in resistive forces.

Without anisotropic force response, reciprocating burrowers would not advance, instead oscillating in place. In nature, active anisotropy mechanisms exist, such as protruding anchors in earthworm segments (Quillin, 1999) and razor clam legs (Winter et al., 2014), as well as passive anisotropy force mechanisms. Passive anisotropy typically involves skin features, like in the ovipositors of wood wasps (Vincent and King, 1995) and locusts (Vincent, 1976), lizard skin for undulatory swimming in sand (Baumgartner et al., 2007; Maladen et al., 2011), and snake scales with sidewinding motion (Hu et al., 2009; Marvi et al., 2014). When implementing actively controlled anisotropy in robotic applications, the robot may require an additional actuator to control anchors, as demonstrated in (Winter et al., 2014; Huang and Tao, 2022; Zhong et al., 2023), or fine-tuning of control inputs for vibro-impact actuators (Barenboim and Degani, 2020). Passively controlled anisotropy on the other hand does not necessitate additional appendage actuation, just internal body movement.

In the existing literature, reciprocating burrowing robots often use passive anisotropy through a range of soft or flexible mechanisms, such as kirigami skin (Liu et al., 2019; Huang and Tao, 2020), setae-inspired flexible structures (Drotman et al., 2022), or even the natural anisotropy of stress states in soil when moving vertically upward (Tao et al., 2020). To assess their burrowing translation ratio, we define it as the net forward displacement over the total linear stroke length:

$$R_{T}=\frac{net\;propulsion}{total\;stroke}=\frac{D_{b}}{D_{s}}.$$ (1)

where *D*_*b*_ represents the distance traveled in the desired direction, and *D*_*s*_ denotes the total stroke of linear actuation. In previous studies focusing on horizontal reciprocating burrowers with passive anisotropy mechanisms, without actuated appendages, referenced in Table 1, locomotion translation ratios range widely. Cases with higher *R*_*T*_ are observed moving in the upward direction, out of the media. The major challenge to horizontal burrowing motion is the small difference between resistive forces in opposite directions. We do not include the works such as (Chopra et al., 2023) or (Li et al., 2021) in this comparison because they use articulated fins and not linear actuation with passively deployed fins, however we note that they reach 45% and 9% translation ratios, respectively. This is an important distinction because stroke length and appendage size are inherently coupled when burrowing is achieved through direct appendage actuation. For linear actuation with passive appendages, stroke length and fin geometry can be independently designed. We are also not comparing efficiency to other modalities of burrowing, such as growing, drilling, excavating, etc. We look only at reciprocating asymmetric burrowers. Burrowing differs from surface locomotion, for which appendages can be removed from the media. For example, in the case of flipper-based locomotion robot (Mazouchova et al., 2013), the machine can achieve near 100% *R*_*T*_, as defined in the current paper, since the forward strokes of the flippers experience no backward resistance as they move through air. On the other hand, burrowing machines experience both forward and backward appendage motion resistance.

**TABLE 1:** Performance of reciprocating burrowing robots without actuated appendages.

*Robot*	*Burrowing Direction*	*Media Type*	*Translation ratio* (*R*_*T*_, %)	*Average speed*
Kirigami skin soft robot Huang et al. (2020) ^b^	Upward (guided)	Sand (Ottawa F65)	15−75^a^	4.5 mm/s
Razor clam inspired burrower Tao et al. (2020) ^b^	Upward (guided)	Sand	20–70^a^	7.7 mm/s
Soft digging worm Drotman et al. (2022)	Horizontal	Sand	15−25^a^	0.2 mm/s
Origami feet burrower (Present work)^b^	Horizontal (guided)	Glass beads	46.8	7.8 mm/s

a Performance estimated from given trajectory curves.

b Robot movement is constrained by a railing.

While these prior works have involved deploying structures and other mechanisms for creating anisotropy, few have utilized concepts from origami. For robotic applications, origami is a morphological approach to creating deployable structures that can regulate force response to the surrounding media, and has been used in various robotic locomotion applications, such as crawling (Rafsanjani et al., 2018), swimming (Sharifzadeh et al., 2021), and generating thrust (Sharifzadeh and Aukes, 2020). Deployable origami is promising for burrowing since it can generate large deformations in both volume and cross-sectional area, affecting the resistive force of the structure. Recent explorations of origami for burrowing include compliant fins (Li et al., 2021) or setae (Drotman et al., 2022), which enable horizontal burrowing, while kirigami skin (Huang and Tao, 2020) shows promise for upward burrowing. These foldable structures have shown passive anisotropic forces. However, study of design parameters and fabrication using laminate structures for improving translation ratio is limited.

Engineering origami often exploits multiple materials for the structure, such as laminate structures (Wood et al., 2008), therefore designers can tune the mechanical characteristics of the structure by the patterning of the material. The ratio between joint and facet and selective patterning of those elements provide additional design parameters for engineering origami, such as flexible facet design (Lee et al., 2017) to ease kinematic constraints, or adjustable width joint (Kim et al., 2020) for regulating bi-stability of the morphing structures. In the current work, we utilize both the fabrication methods and patterning design characteristic of origami. Although we apply smart composite laminate origami fabrication methods only to a single degree of freedom folding joint, we test how the details of this joint design interact with granular media. This work may therefore prove relevant for informing the implementation of higher complexity origami structures developed for future underground applications.

### 1.1 Overview

**FIGURE 1:**
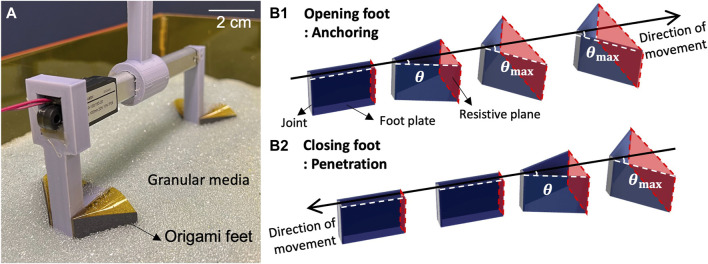
**(A)** Reciprocatng burrower **(B1)** Sequence of foot opening for anchoring in the media **(B2)** Sequence of foot closing for penetration through the media.

In this paper, we study a passive origami foot for anisotropic resistive force, which we parametrically vary to understand effects on burrowing performance. We present the horizontal burrowing robot illustrated in Figure 1A, which transfers pure reciprocating linear motion into directed burrowing motion using a pair of passive origami feet. During actuation, if a pair of feet are translated in the “opening foot” direction, as illustrated in Figure 1B1 , the interaction with the media causes the feet to open until they reach a maximum angle. Conversely, dragging a pair of feet in the “closing foot” direction causes them to close, as shown in Figure 1B2. Granular Resistive Force Theory (RFT) indicates that cross sectional area is a major factor in the drag force a body experiences when moving through granular media (Li et al., 2013). As the feet open and close during motion, they passively alter their cross sectional areas; this area is called the resistive plane for a given foot pair. The difference in resistive plane area between the front and back feet of the burrower results in net forward motion. As in Table 1, our approach achieves burrowing *R*_*T*_ of up to 46.8%. In Section 2, we present the robotic hardware examined in this work, including both individual foot fabrication and the integration of multiple feet into the reciprocating system.

We hypothesize that the locomotor translation ratio of this burrower can be improved by increasing the anisotropy of its passive appendages. We test this by altering the maximum foot opening angle *θ*_*max*_, as well as the details of flexible joint fabrication. We also hypothesize that a change in net foot anisotropy should be accompanied by a change in stroke length, to mitigate inefficiencies that emerge during the cyclical opening and closing of the feet. We vary stroke length to understand the role of contraction-expansion foot state transitions on overall *R*_*T*_.

In Section 3, we describe the modeling and experimental methods used to characterize the deployment and resistive forces of a foot, as well as the translation of these behaviors to reciprocating locomotion. We then present model results of work done by the origami feet as a function of design parameters in Section 4. The modeling techniques used in this work are intended to be used as design tools rather than direct models. Section 5 discusses the relationship between observed individual foot behavior and overall locomotion ability, compares model and experimental results, and discusses study limitations and directions for future work.

## 2 Robotic components and system

### 2.1 Passively deploying origami feet

**FIGURE 2:**
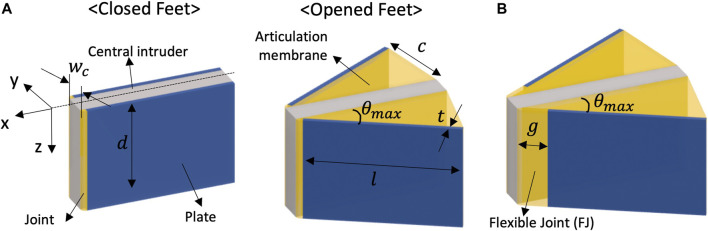
Schematics of a pair of feet **(A)** Components and parameters of folding feet in closed and opened configurations **(B)** opened feet with the flexible joint design.

Figure 2A shows the geometric details of two origami feet in both closed and opened states. Each foot is comprised of a single folding joint, a folding plate that rotates about it, and a plate mounted to a rigid central intruder of width *w*_*c*_. Two feet are attached to the central intruder, one on each side, allowing the feet to independently but symmetrically deploy. Without this rigid central intruder to constrain overall foot orientation, the structure tends to open asymmetrically and twist to reduce drag. The axis of the folding joint is oriented parallel to the gravity direction $\left(\hat{z}\right)$ , and the penetrating direction of the foot translates horizontally in the forward direction of propulsion $\left(\hat{x}\right)$ . When the foot is dragged in the anchoring direction $\left(-\hat{x}\right)$ , the interaction with the media causes the foot to open. As the plate unfolds, the relative angle between the central intruder and the plate form the foot angle *θ*). An articulation membrane acts as the geometric constraint for limiting the maximum folding angle of the foot to *θ*_*max*_. Width of this membrane is altered in the present study to change the maximum reachable angle, all of which are < 90°.

For folding structures in granular media, granular particle jamming and joint stiffening can present challenges for the deployment of small origami mechanisms. In a folding joint with a narrow fold line, such as in Figure 2A, particles can get stuck between the two plates, preventing the foot from closing. To avoid jamming, additional flexible joint (FJ) material is introduced into the origami foot, as shown in Figure 2B. By creating a wide fold line with a margin *g*, this flexible joint allows for multiple particles to remain between the plates even when it is fully closed.

We compare feet both with and without the FJ design to understand the effect of this origami fabrication detail on locomotion performance. We fix the scale of the foot by making the length *l* = 30 *mm*, the depth of the joint and central intruder as *d* = 20 *mm*, and the thickness of the foot as *t* = 1 *mm*. Width of the flexible joint is *g* = 5 *mm*, while the regular joint width is 1 mm. Edge length of the articulation membrane *c* is determined by the maximum foot angle (*θ*_*max*_).

**FIGURE 3:**
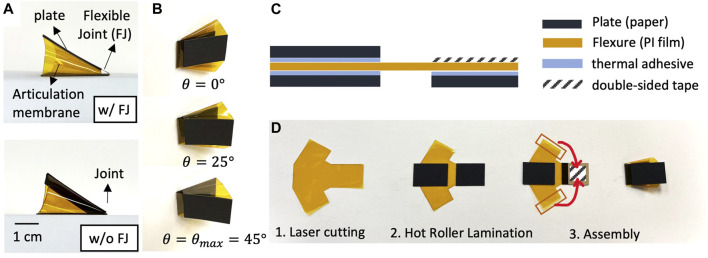
Foot prototype and fabrication method **(A)** Side view of assembled origami foot with and without the flexible joint (FJ) **(B)** Foot shape in three different folding angles **(C)** Side view of foot laminate **(D)** Assembly process.

The primary foot elements of are shown as real assemblies in Figure 3A. Figure 3B shows one prototype across different *θ* angles, as well as how the articulation membrane constrains its maximum foot angle. A schematic of the laminate is shown in Figure 3C. The other side of the structure, which is later connected to the burrower body, is single sided laminate. Fabrication of the foot is based on a two dimensional (2D) fabrication process followed by three dimensional (3D) assembly by hand (Figure 3D). First, we prepare the materials and patterned geometry. Thick paper board (Pacon Railroad Board, 100 um) is used as the plates, and Polyimide (PI) film (Kapton, 25 um) is used as the flexure. For the adhesion between plate and flexure, we use thermally-activated sheet adhesive (GBC Octiva Hot Mount, 17.5 *μm*) applied to the flexure material before patterning. Then, we pattern the plates and flexure by laser cutting (VersaLaser, VL-200). Second, the sandwich composite is created using hot roller lamination. Cut plates are visually aligned on each side of the flexure and a hot roller laminator (Cheminstruments, HL-100) is used to adhere the plates to the flexure by activating thermal adhesive. The laminator is used with a roller temperature of 140°*C* and a nip pressure of 20 psi. Finally, we assemble the foot by joining the sides of the PI film with double-sided tape (3M 468 MP Adhesive Transfer Tape Sheets).

### 2.2 Reciprocating burrower

**FIGURE 4:**
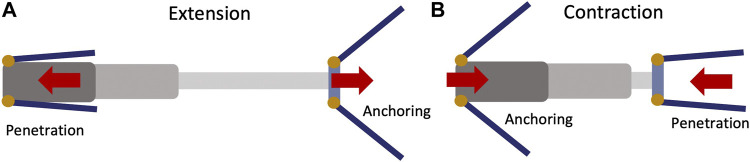
(Top view) Schematics of burrowing propulsion by passive deployment triggered by linear reciprocating motion. **(A)** When body extends, the front feet penetrate and the rear feet anchor in the media, resulting in front foot motion. **(B)** When the body contracts, the front feet anchor and the rear feet penetrate the media, resulting in rear foot motion.

As illustrated in Figure 4, the reciprocating burrower has two pairs of origami feet, one at the front and one at the rear of the body. A linear actuator links the front and rear body segments, enabling repetitive contraction and extension between the foot pairs. During extension, the front feet penetrate the media while the rear feet anchor, resulting in the front feet moving forward (Figure 4A). During contraction, the reverse should occur, in which the front feet anchor in the media while the rear feet penetrate, resulting in the rear feet moving forward (Figure 4B). This cycle of extension and contraction is repeated, resulting in net forward locomotion. With 100% *R*_*T*_, one locomotion cycle would result in one stroke length of forward travel. In actuality, there are phases in which both the font and back of the robot move simultaneously (neither is fully anchored) while the feet open and close; this will occur when transitioning between the two extension/contraction phases and produce locomotion inefficiency.

**FIGURE 5:**
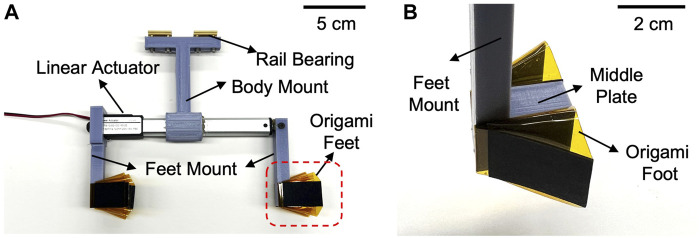
Prototype of reciprocating burrower **(A)** Side view of burrower which has a pair of origami feet on the front and rear of the body. **(B)** A pair of feet is adhered to each side of the middle plate and connected to the linear actuator via foot mount.

The reciprocating burrower hardware prototype is depicted in detail in Figure 5, showing the linear actuator, feet/body mounts (3D printed by Ultimaker 3), rail bearing, and fabricated origami feet. The linear actuator (DC House, Electric Micro Linear Actuator) not only generates the reciprocating motion, but also forms the body of the burrowing machine. Because the propulsive force generated is sensitive to body orientation and substrate properties, the movement of the burrower is constrained to translate along an axis parallel to the direction of propulsion. The body mount runs along a linear guide (drylin^®^ N guide rail, 17 mm) with rail bearings (IGUS NW-02–17 DryLin Miniature Guide Carriage). During testing, only the feet are submerged in the granular media, as constrained by the height of the linear guide and burrower robot dimensions relative to the surface of the media. To actuate the burrowing machine, 12 V DC voltage provided from a power supply and breaker (DaierTek, Reversing Polarity Power Toggle Switch) is used to alter the extension and contraction of the linear actuator for cyclic reciprocating motion.

To facilitate experimentation, the linear actuator and/or feet of this burrowing machine can be easily swapped between trials. The linear actuator can be replaced with one of a different stroke length, while the adhesive tape attaching the feet to the middle plate can be removed to enable new foot attachments.

## 3 Experimental and modeling methods

### 3.1 Experimentation of single foot anisotropy

We investigate the effect of two independent foot design parameters—maximum foot angle and the presence of a flexible joint—on the motions and resistive forces of a single foot. We conduct experiments with six different cases, including three different maximum foot angles (*θ*_*max*_ = 30°, 45°, 60°) for both the cases with and without a flexible joint. We use a mixture of 1 mm and 2 mm diameter glass beads of 1.43 *g*/*cm* ^3^ density as the granular media for these characterization tests.

**FIGURE 6:**
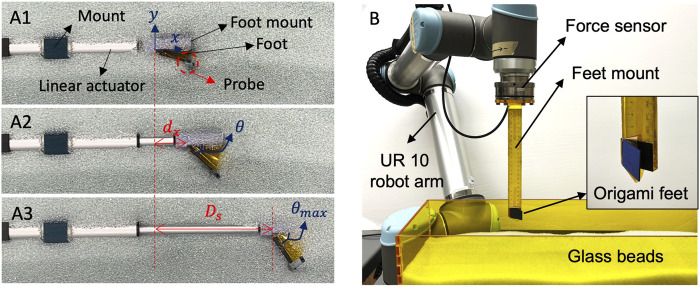
Experimental setup for single foot behavior **(A)** Tracking the deployment profile of a submerged foot **(B)** Experimental setup for measuring resistive force profile of the feet.

We first experimentally analyze the trajectory of the burrower’s robot’s foot as it passively deploys in response to controlled linear actuation. The experimental setup is shown in Figure 6A. While the real burrowing experiment is conducted with the foot fully submerged in the granular media, for the purposes of visualization, a photograph of the experimental setup is provided with the foot at a half-submerged position. A vertical probe is attached at the end of the foot plate, which is exposed after the foot is fully submerged in media for motion tracking. Joint location of the foot when the actuator is fully retracted is set as the coordinate origin (Figure 6A1). As the linear actuator expands and retracts, we record the position of the probe as (*d*_*x*_, *d*_*y*_) over five cycles (Figure 6A2) with a stroke length of *D*_*s*_ = 100 mm (Figure 6A3). The trajectory is calculated using the Tracker video analysis and modeling tool (Brown and Cox, 2009).

We then measure the resistive force profile of the foot during reciprocating motion to quantify its force anisotropy. The experimental setup is shown in Figure 6B. A 6 degree of freedom robot arm (universal Robots, UR-10) equipped with a wrist F/T sensor (ATI, Axia80, sampling rate 150 Hz) measures the $\hat{x}$ force component throughout foot translation. The feet are submerged to 40 mm depth (depth of the vertical center of the foot) and tested over 5 cycles of reciprocating motion.

### 3.2 Experimentation of reciprocating burrowing

**FIGURE 7:**
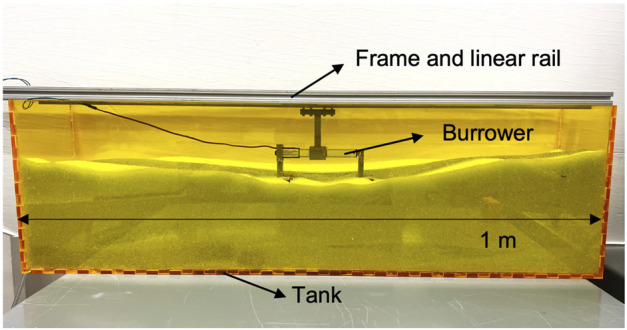
Granular bed for reciprocating burrower testing, with the burrower exposed for visualization.

To evaluate locomotive performance—across both different foot designs and different stroke lengths—we conduct a series of experiments in a granular bed (length = 1 m, width = 18 cm, depth = 30 cm), again filled with glass beads (mixture of 1 mm and 2 mm diameter, density: 1.43 *g*/*cm* ^3^), as illustrated in Figure 7. The linear guide rail for burrower movement is mounted to a stiff aluminum frame fixtured to the side wall of the tank. As described in 2.2, the burrowing machine is attached to the linear guide, allowing for constrained movement along the horizo1ntal axis. Prior to each experiment, the media is leveled to approximate uniform burrowing depth. Feet are then submerged in the granular media at 5 cm depth, and the burrowing machine is driven with cyclic reciprocating motion generated by the linear actuator. We measure the burrowing motion by video in order to estimate *R*_*T*_ of locomotion as defined in Eq. 1. Tests are performed across 1) six different foot designs used in the 3.1 single foot experiments, and 2) two different stroke lengths, 30 mm and 100 mm. The trajectory is again recorded and analyzed using the Tracker video analysis and modeling tool (Brown and Cox, 2009).

### 3.3 Modeling anisotropic foot behaviors

This work explores the utility of granular Resistive Force Theory (RFT) to estimate the forces exerted by the passively deployed origami feet. As a reduced-order model of drag force in granular media, this method provides rapid predictions useful in parametric design studies Treers et al. (2021). This modeling tool is coupled with experimentally measured plate motions from the single foot tests in 3.1 to inform predictions of net work done. We later compare RFT predictions with trends in *R*_*T*_ during real locomotion, but RFT in its current form is not a predictive model of full burrower robot locomotion. We also compare the motion and force predictions from the proposed empirical RFT formulation with Discrete Element Method (DEM) simulations.

#### 3.3.1 Reduced order model for horizontal burrower

Experimental measurements from 3.1 provide plate rotation during both anchoring and penetrating motions. We first propose a model for foot closing as an exponential fit to the mean of the experimental profiles. The proposed geometric model for the opening of a foot is a quadratic fit, where the foot angular position saturates once it reaches *θ*_*max*_. Thus, the relationships proposed for foot angle are: *θ*_*open*_ = *O*_1_*x ^2^ + *O*_2_*x + *O*_3_ and $\theta_{\mathrm{close}}=C_{1}*e^{\left(C_{2}*x\right)}+C_{3}*e^{\left(C_{4}*x\right)}$ , where x is foot displacement from initial state, and *O*_*i*_ and *C*_*i*_ are fit coefficients.

**FIGURE 8:**
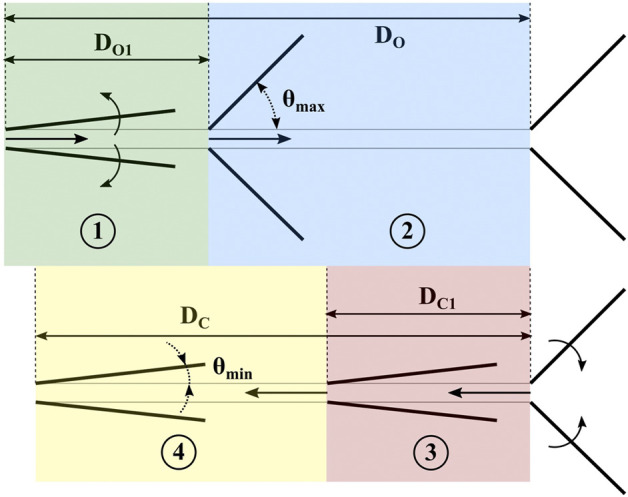
The four stages of proposed model for foot displacement, and relevant geometric parameters.

To estimate resistive force, we break the RFT model into four discrete stages, as shown in Figure 8: two for opening and two for closing. We introduce the parameters *D*_*C*_ and *D*_*O*_, which represent the distances that the feet travel in the closing and opening directions, respectively. *D*_*C*1_ and *D*_*O*1_ here represent the distances the feet require to fully close from a fully opened position, and *vice versa*.

In the “opening” stages 1) and 2), we assume that the effective contact area in RFT is a vertical plane formed by the leading edges of the feet. In the “closing” stages 3) and 4), we assume traditional RFT computation, and use horizontal RFT coefficients *f*_1_ and *f*_23_ as presented in (Treers et al., 2021), where *f*_1_ represents the component of force along the plate surface and *f*_23_ represents the force parallel to the plate normal. The data follows the form $f_{1}=\left(A_{1}*\left(\tanh\left(A_{2}\sin\left(\theta \right)-A_{3}\right)+A_{4}\right)\right)/|{\tilde{F}}_{1}|$ and $f_{23}=\left(B_{1}*\left(atanh\left(B_{2}\cos\left(\theta \right)-B_{3}\right)+B_{4}\right)\right)/|{\tilde{F}}_{23}|$ , where *A*_*i*_ and *B*_*i*_ are fitting terms, and $\left|{\tilde{F}}_{1}\right|$ and $\left|{\tilde{F}}_{23}\right|$ are the force magnitudes recorded at the extremes, i.e., *θ* = 0 and *π*/2, respectively. Fit coefficients, as well as and $\left|{\tilde{F}}_{1}\right|$ and $\left|{\tilde{F}}_{23}\right|$ , are reported in (Treers et al., 2021).^1^ The resulting equations for resistive force (*RF*) in (1)-(4) are thus:

$$RF_{1}=hz\alpha_{x}\left(2l\;\sin\left(\theta_{\mathrm{open}}\right)+w_{c}\right)$$ (2)

$$RF_{2}=hz\alpha_{x}\left(2l\;\sin\left(\theta_{\max}\right)+w_{c}\right)$$ (3)

$$RF_{3}=2*\left({\hat{F}}_{1}\;\sin\left(\theta_{\mathrm{close}}\right)+{\hat{F}}_{23}\;\cos\left(\theta_{\mathrm{close}}\right)\right)+w_{c}hz\alpha_{x}$$ (4)

$$RF_{4}=2*\left({\hat{F}}_{1}\;\sin\left(\theta_{\min}\right)+{\hat{F}}_{23}\;\cos\left(\theta_{\min}\right)\right)+w_{c}hz\alpha_{x}$$ (5)

where ${\hat{F}}_{1}=zlhf_{1}\alpha_{x}\left(\left|{\tilde{F}}_{1}|/|{\tilde{F}}_{23}\right|\right)$ and ${\hat{F}}_{23}=zlhf_{23}\alpha_{x}$ . Depth *z*, length *l*, and height *h* are foot dimensions and *α*_*x*_ is the horizontal resistive coefficient predicted by 2D RFT for a plate oriented vertically and translated horizontally (Li et al., 2013). Angle variables *θ*_*close*_ and *θ*_*open*_ represent changing foot angle throughout the burrowing stage, whereas *θ*_*min*_ and *θ*_*max*_ represent fixed parameters. The empirical model is used to predict net forward work done by a single foot across a full actuation cycle by integrating force with linear actuator displacement. We assume that both expansion and contraction cycles occur with equal foot displacements.

#### 3.3.2 DEM granular bed simulation

We set up a granular bed simulation using the Project Chrono Physics Engine and the NSC complementarity collision model for grains. We choose a bed size of 20 cm length × 10 cm width × 10 cm height, friction coefficient of 0.73, and assume zero cohesion in the media. Grain size is set to 2 mm diameter spherical particles to reduce the number of particles necessary and reduce the computational time. Friction coefficient is derived by testing the value for *μ* which results in a friction angle of 24.6°, using a tilt bed test in the Chrono simulation environment. Density of the particles is 1930 kg/m^3^ from the bulk density of the media, assuming an optimal void fraction *e*_*min*_ of 0.35 for spherical particles. We simulate a system with 10,000 particles, which results in a bed depth of approximately 3.5 cm.

The simulation of the passive origami feet uses a central rectangular intruder body of width *w*_*c*_ = 5 *mm* and, following the foot dimensions illustrated on Figure 2, d = 2 cm, l = 3 cm and t = 1 mm for two feet. Body density is 470 kg/m^3^, and the two plates are free to rotate about their axes, colinear with the central intruder edges. The horizontal distance between the axes of rotation of each foot is defined by the width of the central intruder, *w*_*c*_, plus an extra gap distance between the foot edge and the central intruder on each side to emulate the effect of the flexible joint *g* = 2 *mm*. The feet are initialized at a depth such that the top edges of both feet are aligned with the substrate surface. The central intruder translates horizontally in the media at a velocity of 20 mm/s for a total displacement of 10 cm. For opening trials, once the plate reaches its maximum angle (*θ*_*max*_) the foot is modeled as a rigid body. We average 4 simulations that output translation distance, angular displacement *θ*, and forces experienced by the feet.

## 4 Results

### 4.1 Passive single foot behaviors

**FIGURE 9:**
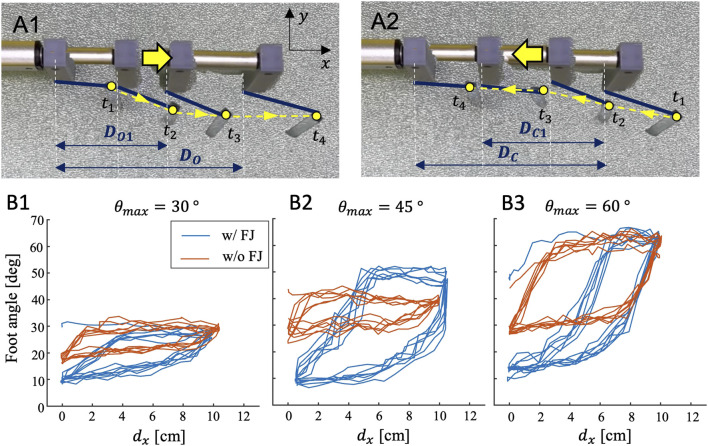
**(A)** Variation in foot deployment profile based on foot parameters for a 100 mm stroke length. **(A1)** Tip trajectory of opening foot **(A2)** Tip trajectory of closing foot. **(B)** Tip trajectory of foot with different *θ*_*max*_: **(B1)** *θ*_*max*_ = 30° **(B2)** *θ*_*max*_ = 45° **(B3)** *θ*_*max*_ = 60°.

Figure 9A shows the tip trajectories of the origami foot with *θ*_*max*_ = 45° and with a flexible joint. The foot angle during opening (Figure 9A1) shows a rapid increase during the initial stages of the stroke (*t*_1_-*t*_2_). It then reaches saturation at its maximum foot angle (*t*_3_), and maintains this configuration until the end of the stroke (*t*_4_). Conversely, the foot angle during closing (Figure 9A2) decreases during the initial stages of forward stroke (*t*_1_-*t*_2_), reaching saturation as the plate folds towards the central intruder. The foot angle appears to reach its steady-state minimum angle at (*t*_3_), and maintains this configuration until the end of the stroke (*t*_4_). The motion profile of the foot is therefore asymmetric during one full stroke.

To quantify the motion anisotropy across foot designs, we compare estimated foot angle across feet with different *θ*_*max*_ (30°, 45°, and 60°), both with and without the flexible joint (FJ). Figures 9B1–B3 show the resultant foot angle over 5 cycles for each foot design. Feet with flexible joints (shown in blue) exhibit a larger range of *θ* values compared to feet without the flexible joint (shown in orange). Across all three *θ*_*max*_ designs, the flexible joint results in a smaller value for *θ*_*min*_ that approaches 10°. In contrast, cases without the FJ result in *θ*_*min*_ values of 20° for *θ*_*max*_ = 30°, and *θ*_*min*_ = 30° for both *θ*_*max*_ = 45° and 60°. We attribute this effect to granular jamming, which is avoided by introducing the FJ. When comparing FJ designs across the different maximum angles (*θ*_*max*_), the angle change during opening/closing becomes steeper as *θ*_*max*_ increases. It also takes more displacement, *d*_*x*_, to reach saturation of foot configuration for full opening and closing. Therefore, the area inside the displacement curve is greatest for feet with an intermediate value of *θ*_*max*_ (= 45°). Assuming that this angle is closely related to the drag force applied by the foot, we anticipate that the *θ*_*max*_ = 45° feet with FJ will demonstrate the largest anisotropy and best burrowing performance. Interestingly, for the *θ*_*max*_ = 60° case (Figure 9B3) only, the feet without the FJ result in a larger displacement curve area than those with FJ feet.

### 4.2 Passive deployment of foot by DEM

We confirm these trends in Chrono granular simulations. We show the foot angular position *θ*, as a function of horizontal displacement, for three different values of the ratio between gap size *δc* and grain size d. Notably, the no gap case (*δc* = 0) resulted in a longer translation (approximately 10 additional mm) to reach steady-state “closed” position, and its closed position *θ*_*min*_ is several degrees larger than that when a non-zero gap is included in the model. This behavior reflects what we observe experimentally, in Figure 9 the average *θ*_*min*_ for cases with FJ was 10–20 deg less than those without. Due to non-rigidity in links in the simulation structure, *θ*_*min*_ in simulation reaches lower values than that in the physical system.

**FIGURE 10:**
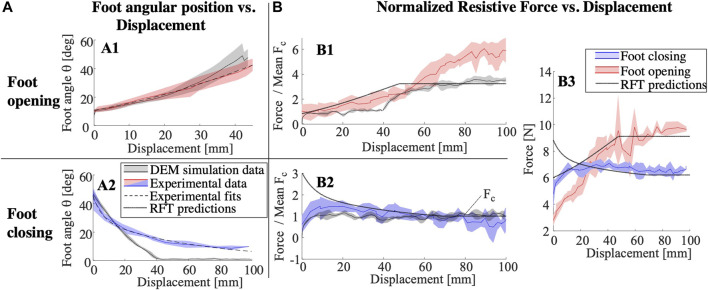
**(A1)** Foot angle as a function of foot displacement for foot opening. Chrono simulation results shown in black, and experimental data shown in red, along with parabolic fit to the data. **(A2)** Foot angle as a function of foot displacement for foot closing. **(B1)** Resistive force, scaled to mean closing force, as a function of displacement, for foot opening. Chrono simulation results shown in black, and experimental data shown in red, along with RFT model-predicted force (black dashes). **(B2)** Resistive force, scaled to mean closing force, as a function of displacement, for foot closing. **(B3)** Resistive force for full foot and center bar assembly, as a function of displacement, for both experiments and RFT predictions.

Angular position over displacement is shown in Figures 10A1, A2 for the “opening” and “closing” phases of foot motion, respectively, as defined in Figure 8. Experimental results, empirically fit curves, and Chrono simulation results are all depicted. In simulation, we find that it takes approximately 40 mm to reach fully closed or fully open states. However, in experiment, it takes 47 mm to reach a fully “open” state at 45°, while it takes approximately 90 mm to reach the fully “closed” state at 10°. This delayed response in the experimental system is likely due to a combination of additional damping in the media, granular jamming, deformation of the burrower structure and other non-idealities not captured in this DEM simulation.

Resultant RFT-predicted forces for a single foot as a function of displacement are shown in Figures 10B1, B2, and compared with results obtained from both experiments and DEM simulations. In order to present experimental data for a single foot, we subtract measured force contributions of the central intruder from the net measured force data and the forces are normalized to the mean force obtained in the fully closed position, *F*_*c*_. In the opening trials (B1), the experimental results match the RFT model until 40–50 mm of displacement, after which the measured forces continue growing, likely due to mounding effects at the media surface. While the DEM simulation captures this gradual increase in force with mounding, it also underestimates the overall force at steady state as compared with experiments. In the closing trials (B2), the RFT-predicted forces start substantially higher than the DEM and experimental results, before converging at about 20 mm. This is likely because the nature of granular interaction at the transition point is not well-described by RFT; changes in grain compaction are not captured as a body re-interacts with media upon loading reversal. Figure 10B3 represents the RFT model force magnitudes taking into account the force from two feet and the central intruder, as compared with experimental data for the entire foot pair assembly. The steady state forces predictions appear accurate while we see substantial errors at less than 20 mm of displacement.

**FIGURE 11:**
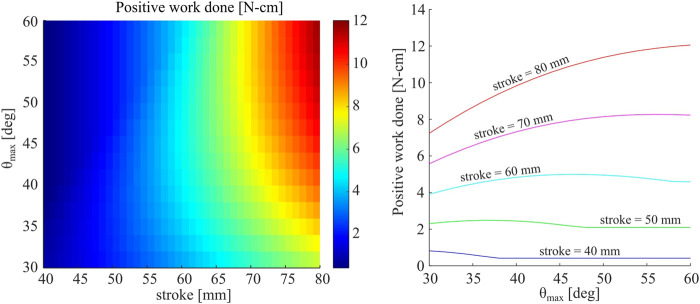
(Left) Net positive work done by a single foot for a single cycle, including both opening and closing stages, as predicted by the proposed RFT model. Work is plotted as a function of both maximum angle of foot opening, *θ*_*max*_, as well as total stroke. **(**Right) Net work plotted as a function of *θ*_*max*_ for five different values of stroke.

We assume *D*_*O*_ = *D*_*C*_, and plot the resultant net work done during a full expansion and contraction cycle for a single foot with a FJ, as estimated by the empirically-fit RFT model. Results from this parametric study, varying stroke length and *θ*_*max*_, are shown in Figure 11. We observe that longer strokes and larger maximum open angles *θ*_*max*_ produce higher positive work values. However, for a given stroke length, an intermediate value of *θ*_*max*_ appears to maximize positive work. For example, a stroke of 60 mm results in an optimum *θ*_*max*_ of ≈ 47°. Feet with lower values for *θ*_*max*_ require less displacement to open fully, thus reach maximum resistive force more quickly. On the other hand, while larger values of *θ*_*max*_ will increase the maximum resistive force achieved, this is at the cost of reaching steady-state more gradually. Therefore, we find an intermediate region at which opening speed is traded off with maximum thrust. Shorter strokes cannot articulate the full opening and closing motions required to benefit from larger values of *θ*_*max*_. Conversely, systems with longer strokes will generally benefit from larger values of *θ*_*max*_.

### 4.3 Burrower demonstration and locomotion performance

**FIGURE 12:**
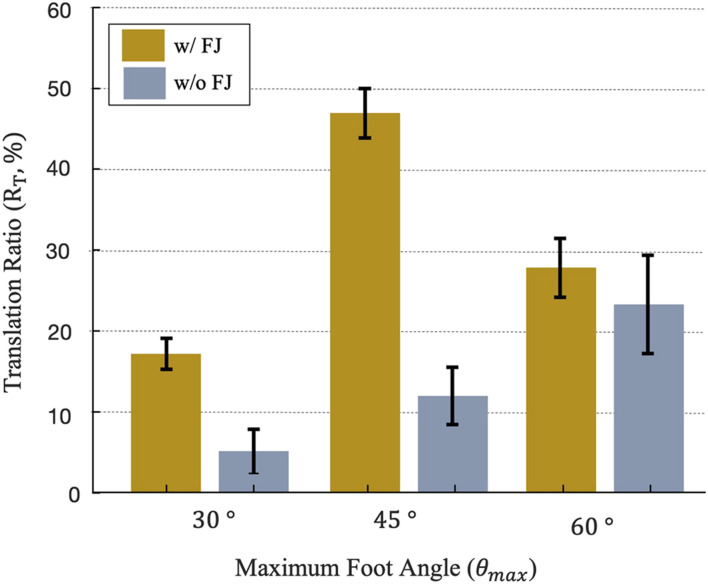
Translation ratio of a 100 mm stroke length reciprocating burrower across 6 foot designs, averaged over 10 strokes, with error bars indicating the standard error.

Figure 12 depicts the translation ratio of the complete reciprocating burrowing robot with a 100 mm stroke linear actuator and six different feet, as described in 3.2. The mean and standard error in burrowing *R*_*T*_ is plotted for 10 strokes, or two sets of 5 continuous strokes, for each foot design. As predicted from trajectory analysis in 4.1, the burrower with the *θ*_*max*_ = 45° and flexible joint feet shows the greatest locomotion translation ratio. Note that the RFT model, as depicted in Figure 11, implies that *θ*_*max*_ = 60° feet would provide higher positive work than *θ*_*max*_ = 45° feet for strokes larger than 70 mm. However, free locomotion differs from the motion-constrained RFT model: due to movement of both the front and back feet during a single extension or contraction event, traveling displacement for the anchoring feet (*D*_*O*_) is not equal with the closing displacement of the penetrating feet (*D*_*C*_) and each individual foot pair travels < 100 mm overall. As a result, positive work estimation of single foot (Figure 11) overestimates the optimal *θ*_*max*_ for the free burrowing locomotor with multiple feet. For all three different *θ*_*max*_ values, feet with flexible joints provide larger efficiencies than those without flexible joints. Also, feet without flexible joints show larger deviation in *R*_*T*_, potentially due to the stochasticity of granular jamming.

**FIGURE 13:**
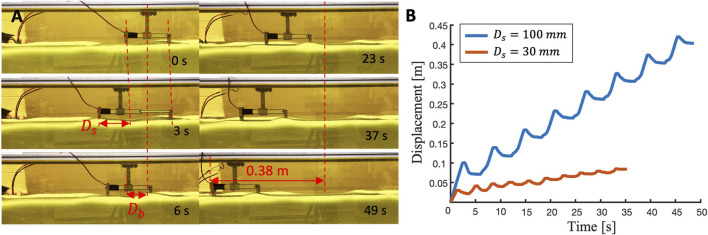
Performance of a reciprocating burrower with *θ*_*max*_ = 45° with flexible joint feet **(A)** Locomotion of burrower with longer stroke (*D*_*s*_ = 100 mm) **(B)** Burrower displacement plot of burrower with different strokes (*D*_*s*_ = 100 mm and 30 mm).

Burrowing performances are shown in Supplementary Video S1. Figure 13 shows the detailed performance of the reciprocating burrower with the most efficient foot design (*θ*_*max*_ = 45° with FJ). Figure 13A shows the intermediate steps of burrowing with a stroke length *D*_*s*_ = 100 mm. While the linear actuator extends with its stroke *D*_*s*_ (3 s), the front feet penetrate forward while the rear feet retract backwards. Next, when the linear actuator contracts, the front feet retract while the rear feet propel forward. After one cycle of reciprocating motion, the robot’s net forward motion is *D*_*b*_ (6 s). As this cyclic actuation is repeated, the robot continues to locomote forward. After 8 cycles of reciprocating motion with the average period of 6.1 s, the robot has traveled 0.383 m along the horizontal axis with 46.8% of *R*_*T*_ and average speed of 7.8 mm/s. The power consumption of the burrower is 1.32 W.

To evaluate the effect of stroke length on burrowing translation ratio, we performed identical experiments but with a different linear actuator with a shorter stroke length (*D*_*s*_ = 30 mm). As shown in Figure 13B, the robot driven by the shorter stroke actuator travels only 0.083 m along the horizontal axis with 26.8 % of *R*_*T*_ and average speed of 2.3 mm/s (average period is 3.5 s). Therefore, the longer stroke leads to faster burrowing with greater translation ratio for this particular foot design.

## 5 Discussion

In this work, we present a reciprocating burrowing robot with horizontal translation ratio up to 46%, achieved through the parametric design of origami feet. We define translation ratio as the ratio of net propulsion displacement to the total actuator stroke, representing the portion of symmetric cyclical movement that translates into desired asymmetric motion. The enabling feet, passively-deployable structures placed at the anterior and posterior of the robot, produce shape change triggered by interaction with the media. Overall, longer stroke lengths improve translation ratio. We also find that small details of the origami structure, including both rotational range of motion and flexible joint width, can substantially alter locomotor performance. In fact, there exists an optimal foot design for a given reciprocating stroke length. The hypothesis that locomotor translation ratio is improved by increasing the anisotropy of passive appendages therefore appears to hold true.

This work also uses granular Resistive Force Theory as a method to understand origami foot design trends. RFT estimates forces assuming that motion is known, so in this work we first characterize plate motion with a fit empirical model and then use it to predict foot drag forces. While nonidealities of the granular media and errors in the model limit the accuracy of force predictions, especially at motion transitions, meaningful design trends emerge from parametric study that agree with experimental trends. In the current form, the RFT model is applied for a given linear foot displacement. However, during real burrowing, the true forward and backward movement of the feet will vary. Future work will seek to generate a quasistatic simulation of foot displacements during free locomotion, for example, using granular limit surfaces Huh et al. (2023).

This study has several limitations. The method of creating a driving force with anisotropy is sensitive to the surrounding environment, such as surface mounding. Because successful operation is reliant upon a difference in forces between the front and back feet, slight differences in foot depth alter performance. In this study, we mitigate these factors by constraining the motion of the robot to the one degree-of-freedom railing such that depth and orientation of the robot relative to the media remain similar throughout locomotion. Future work will explore less constrained movement. The robot feet are also made of flexible joints fabricated with adhesive tape, so durability is low. The Kapton film tore and the adhesive tape occasionally detached during experimentation. Stronger materials and assemblies would be needed if larger drag forces were applied in future work.

Although not addressed in this study, other foot design parameters will also influence burrowing performance. For example, we assume in this study that the resistive forces should scale with foot area, according to RFT. However, the ratio between foot length and angular displacement profiles may not be as straightforward. While we expect larger feet will require larger strokes to fully open, further experimentation and simulation may be necessary to determine this relationship. Factors such as the foot length and flexible joint width to grain size ratio, granular friction coefficients, etc. would likely complicate these scaling arguments. Additionally, in this study we assume the forces are proportional to the depth in the media, which may not hold for all types of media. The effect of the width of the central intruder has also not been studied. We assume that the width of the central intruder will not affect the burrowing performance or net work generated; we assume that the width only affects the nominal or baseline level of resistive force. In other words, changes in the width of the central wall would offset the force for both penetration and anchoring. Further experimentation could help validate our assumptions.

The modeling methods introduced here represent major simplifications of the real burrowing system. First, the flexible PI film membrane was not incorporated into either RFT or DEM models, as these methods currently present challenges for simulating these flexible elements. We instead assume that the membrane in our simulations only affects the angle constraint, which ignores some effects which occur due to the folding and buckling of the membrane. Second, the application of RFT models to folding feet is limited by the fact that RFT does not directly predict motion from forces; we rely on experimental fits for angular displacement of the foot. In a recent alternative application of RFT to model underactuated feet (Li et al., 2021), experimentally fit damping coefficient to enable motion prediction and account for damping in the mechanism flexures. We present another approach to RFT-driven models for passively moving feet. Third, we modeled unconstrained foot movements and used it to predict single foot translation ratios. The next step in this line of work should be to build upon this tool to inform predictions of overall robot burrowing behaviors and efficiencies in future work. Last, as the burrower moves slowly, we assume quasistatic motion and do not use dynamic RFT. Therefore, the current model would break down as speed increases.

Regardless of these limitations, this work demonstrates efficient burrowing with a simple long-stroke linear actuator and compact hardware without need of complex controller or sensors. We observe that this performance is sensitive to small variations in the passive deployable foot structures, including both overall motion constraints and the width of the flexible origami joint. We expect that the reciprocal foot design principles observed in this work can translate to other burrowing applications In particular, origami structures benefit from scalability and design flexibility (Belke and Paik, 2017; Filipov et al., 2017), therefore such mechanisms could be tuned for different depths, media types, and robot motions. However, designers should carefully consider the deployment range of motion and the potential for grains to interfere with joint movements to optimize performance.

## Data Availability

The raw data supporting the conclusion of this article will be made available by the authors, without undue reservation.

## Footnotes

1 In this study, *θ* differs from angle definitions in Treers et al. by *θ* = *ψ* − *π*/2, where *ψ* represents plate angle in the horizontal plane in (Treers et al., 2021).

## Supplementary material

SUPPLEMENTARY VIDEO S1 Video summarizing working principle, key component of robot desisn, modeling and experimental results.
